# Microbiomes and Planctomycete diversity in large-scale aquaria habitats

**DOI:** 10.1371/journal.pone.0267881

**Published:** 2022-05-12

**Authors:** Claire E. Elbon, Gary R. LeCleir, Matthew J. Tuttle, Sophie K. Jurgensen, Thomas G. Demas, Christian J. Keller, Tina Stewart, Alison Buchan

**Affiliations:** 1 Department of Microbiology, University of Tennessee, Knoxville, Tennessee, United States of America; 2 Tennessee Aquarium, Chattanooga, Tennessee, United States of America; The University of Akron, UNITED STATES

## Abstract

In commercial large-scale aquaria, controlling levels of nitrogenous compounds is essential for macrofauna health. Naturally occurring bacteria are capable of transforming toxic nitrogen species into their more benign counterparts and play important roles in maintaining aquaria health. Nitrification, the microbially-mediated transformation of ammonium and nitrite to nitrate, is a common and encouraged process for management of both commercial and home aquaria. A potentially competing microbial process that transforms ammonium and nitrite to dinitrogen gas (anaerobic ammonium oxidation [anammox]) is mediated by some bacteria within the phylum Planctomycetes. Anammox has been harnessed for nitrogen removal during wastewater treatment, as the nitrogenous end product is released into the atmosphere rather than in aqueous discharge. Whether anammox bacteria could be similarly utilized in commercial aquaria is an open question. As a first step in assessing the viability of this practice, we (i) characterized microbial communities from water and sand filtration systems for four habitats at the Tennessee Aquarium and (ii) examined the abundance and anammox potential of Planctomycetes using culture-independent approaches. 16S rRNA gene amplicon sequencing revealed distinct, yet stable, microbial communities and the presence of Planctomycetes (~1–15% of library reads) in all sampled habitats. Preliminary metagenomic analyses identified the genetic potential for multiple complete nitrogen metabolism pathways. However, no known genes diagnostic for the anammox reaction were found in this survey. To better understand the diversity of this group of bacteria in these systems, a targeted Planctomycete-specific 16S rRNA gene-based PCR approach was used. This effort recovered amplicons that share <95% 16S rRNA gene sequence identity to previously characterized Planctomycetes, suggesting novel strains within this phylum reside within aquaria.

## Introduction

The importance of microbiomes to the health of their hosts and environments is undisputed [1–3]. Microbiomes play critical roles in ecosystem viability by contributing to nutrient cycling, pollutant remediation, and the stability and health of plant and animal communities [4, 5]. Microbial community dynamics are proposed to be accurate indicators of the health of large-scale systems and their individual residents [6]. In oceans, estuaries, and freshwater habitats, microbial communities are the primary drivers of biogeochemical cycles [7–9] and are catalysts for many essential chemical transformations [10]. While their taxonomic composition differs from natural environments, microbial communities in aquaria are equally important in driving system chemistry and the overall health of their resident fauna [11, 12].

Nitrogen cycling is particularly important in closed-system aquaria. Several nitrogen species (e.g., ammonium and nitrite), derived from the waste products of aquaria macrofauna, are toxic to aquarium inhabitants [13]. Nitrification, a process performed exclusively by a diverse group of bacteria and archaea, removes highly toxic ammonium by first converting it to nitrite then nitrate via two subsequent oxidation reactions [14, 15]. While less toxic than either ammonium or nitrite, at high concentrations nitrate can also be harmful to macrofauna [16]. Furthermore, toxic levels of nitrate can commonly build up in aquaria as the mechanisms for its removal from these generally closed systems are limited. Methods to resolve high levels of nitrate include dilution via water changes and sulfur-driven denitrification (SDN), a chemolithotrophic process by which denitrification is coupled with oxidation of reduced sulfur compounds [17]. However, water changes are time-consuming and costly [11, 17], and SDN can be subject to chemical and membrane fouling [18, 19].

Use of microbes capable of anaerobic ammonium oxidation (anammox) is one possible alternative to mitigate the buildup of toxic nitrogen species in aquaria. The anammox pathway, found exclusively within a subgroup of the Planctomycetes bacterial phylum, can transform nitrite and ammonium to dinitrogen gas and water [20, 21]. Thus, these bacteria are capable of simultaneously resolving build-up of the two most toxic nitrogen species. Anammox-performing Planctomycetes have been reported to co-occur with microbes that carry out various nitrogen cycling processes that yield high local abundances of nitrite and ammonium (i.e., nitrification and ammonification) [22]. These anaerobes are estimated to contribute over 50% of the global nitrogen gas release from oceans. Given the low energetics of the anammox reaction, this type of energy metabolism does not typically support robust growth. As a consequence, these bacteria typically comprise < 1% of the microbial communities in the environments in which they are most active [23, 24]. Successful use of anammox Planctomycetes to transform nitrogen species into the more biologically inert form (N_2_) via bioaugmentation has been implemented in wastewater treatment facilities, where both ammonium and nitrite are plentiful [25, 26].

Understanding the extent to which anammox bacteria are naturally present in large-scale commercial aquaria is a necessary first step in assessing the viability of harnessing this process for nitrogen removal via biostimulation or bioaugmentation. The Tennessee Aquarium in Chattanooga, Tennessee (USA) houses exhibits that vary in salinity, temperature, volume, macrofauna, and filtration rate. This facility provides an opportunity to assess the baseline prevalence of Planctomycetes in closed aquaria systems with distinct chemical and physical properties. Here, we describe a broad-scale microbiome analysis that was coupled with a Planctomycete-specific approach to assess the microbial community composition and dynamics across four distinct exhibits.

## Materials and methods

### Sample collection

Samples for 16S rRNA-based microbial community analysis were collected weekly from the Tennessee Aquarium in Chattanooga, TN for four consecutive weeks during June 2017. Water and sand filtration systems were sampled in triplicate from four different tanks (designated T7, T20, T30, and T34) differing in temperature, salinity, turnover rates and resident macrofauna (Table 1). Water turnover is mediated by large mechanical filtration systems that effectively aerate each of these aquaria. Dissolved oxygen is monitored in all tanks and does not fall below 90% saturation, showing minimal variation (<3%) across depth and time. The water in these tanks is continuously cycled through the filtration systems. These systems do not undergo full water exchanges. For water samples, approximately 1 L of surface collected water was filtered through Sterivex™ cartridges with 0.22 μm membrane filters (Millipore-Sigma, USA). For each sand filtration system, approximately 30 g of sand was collected into Whirl-Paks® bags (Nasco, USA) from drained units using sterilized spoons. Samples for metagenomic analyses were collected from the same set of tanks and also a biological denitrification system attached to tank T30 during a single sampling time point in January 2018. Immediately following collection, all samples were frozen in liquid nitrogen until transferred to the University of Tennessee where they were stored at -80˚C until DNA extractions were conducted.

**Table 1:** Characteristics of Tennessee Aquarium study exhibits.

Tank	Salinity	Temperature(± 0.5°C)	Volume (gallons)	Depth (m)	Turnover rate^a^ (min)	Representative macrofauna
T7	Freshwater	16.5 (Cold)	17,000	2.13	62	Pike and Sturgeon (20 specimens)
T20	Freshwater	25.8 (Warm)	15,000	2.43	60	Cichlids (81 specimens)
T30	Marine^b^	24. 8 (Warm)	620,000	9.75	132	Sharks, sea turtles, reef fish (>2800 specimens)
T34	Marine^b^	9.5 (Cold)	35,000	1.52	50	Octopus, sea stars and anemones (30 specimens)

^a^ Time it takes the entire volume of water to pass through the filtration systems.

^b^ The saltwater tanks are filled with artificial seawater.

### 16S rRNA gene-based microbial community analysis

DNA was extracted from water and sand samples using a phenol-chloroform protocol. For water samples, the initial steps of the extraction protocol were performed within Sterivex™ cartridges by plugging the outflow port with Cha-seal clay (DWK Life Sciences, USA). Reagents were added to the cartridges directly using a needle and syringe; Luer-lock caps were used to seal the inflow port. For each sand sample, 0.5 g of material was placed in a 15 ml plastic tube (Falcon). To all samples, 1.7 ml CTAB extraction buffer, 65 μL proteinase K (10mg/ml), 65 μL lysosome (10 mg/ml), and 162 μL of filter-sterilized SDS (10% in deionized water) were added. Tubes and cartridges were incubated in a rotary agitator at 65˚C for 2 h. Following incubation, 800 μL aliquots were pipetted into 1.7 ml microcentrifuge tubes and cooled to 4˚C. The remaining solution was stored at -20˚C for later additional extraction of low yield or low-quality samples. An equal volume of phenol:chloroform:isoamyl alcohol (P:C:I, 25:24:1, pH 8.0) was added to each sample and vortexed. The aqueous and organic layers were separated via 10,000 rpm centrifugation for 5 minutes at 4˚C. The aqueous layer was transferred to a new sterile tube. Two additional P:C:I extractions were repeated with the aqueous layer. To the final aqueous layer, 450 μL of 100% isopropanol was added and incubated overnight at room temperature. Tubes were centrifuged at 10,000 rpm for 20 minutes to pellet DNA. After decanting, DNA was washed with 70% ethanol, centrifuged at 10,000 rpm for 5 minutes, and dried for 15 minutes in a laminar flow hood. DNA was then suspended in 50 μL of sterile nuclease-free water at 50˚C. DNA was quantified using a Nano-drop® ND-1000 Spectrometer (Thermo Fisher Scientific, USA) and samples were stored at -80˚C.

16S rRNA genes were amplified using the EarthMicrobiome project PCR primers 515F-Adapt and 806R-Adapt following published protocols (S1 Table [27]). Amplified PCR products were ~270 bp in size, allowing for overlap in paired ends reads [27]. PCR products were sequenced using the MiSeq Illumina® platform at the University of Tennessee’s Genomic Core Facility. The Mothur software package (version 1.39.5) was used to process sequences and remove low quality reads following established criteria [28]. Mothur was also used to cluster sequences into operational taxonomic units (OTUs; at >97% identity to cultured organisms) following the Schloss MiSeq SOP for 16S rRNA genes analysis [28]. The Primer-e software package (version 7) was used to interrogate the relationships between OTUs across samples. Alpha diversity (Shannon-Weiner and Simpson indices) was also calculated using Primer-e. Pairwise Wilcoxon tests were performed to identify significant differences between diversity measures of habitat samples using the R package ggpubr (version 0.2.5; https://CRAN.R-project.org/package=ggpubr). Detection of “biomarker” OTUs, diagnostic of specific aquaria, was performed using a Linear Discriminant Analysis using the LEfSe tool as part of the BioBakery meta’omics analysis environment [29]. This analysis focused on the two parameters (substrate and salinity) contributing most to clustering of communities on the Primer ordination plot. The input data was provided as read counts and an all-vs-all analysis was performed after the substrate (water vs sand) and salinity (fresh vs salt) were collapsed.

### Metagenomic sequencing and analysis

For metagenome analyses, DNA was extracted from 9 samples (water and sand filters from each exhibit and water from the T30 denitrification tank) using the MO-BIO Power Soil® DNA isolation kit following the manufacturer’s guidelines (QIAGEN, Germany). DNA was prepared for metagenomic analyses using the Nextera® XT genome kit (Illumina®, United States) following manufacturer’s protocol with the minor modification of increasing the PCR cycles from 12 to 15 because of low DNA yield in some of the samples. The samples were loaded at 8 pM with 2% PhiX spike-in on an Illumina MiSeq at the University of Tennessee Genomics Core on a v3, 600 cycle flow cell, reading 275 bases paired-end. Paired-end sequencing data was imported into CLC Genomics Workbench (version 20.0.01; QIAGEN, Germany) where reads were assembled into contigs using the default settings of the De Novo Assembly tool. Coding domains were identified using the MetaGeneMark program and annotated with CLC. Individual reads were then mapped back to assembled contigs for quantification of relative abundances of individual genes. The GhostKoala program [30] within the Kyoto Encyclopedia of Genes and Genomes (KEGG; [31]) was used to automatically annotate CDS (coding sequence) regions within each metagenome and highlight specific genes within various functional pathways.

### Planctomycete-specific 16S rRNA gene amplification and analysis

Due to the relatively high proportion of Planctomycete OTUs found in sand filtration samples from T30 and T34, DNA was extracted from June 2017 archived sample material using the MO-BIO Power Soil® DNA isolation kit (QIAGEN, Germany). A Planctomycete-specific, nested 16S rRNA gene PCR approach was employed to examine the diversity of Planctomycete species in the aquaria and probe for Planctomycetes capable of specific metabolic functions (anammox) (S1 Fig). The initial PCR amplification using the Planctomycete-specific primer Pla46F [32] and the universal bacterial primer 1390R (S1 Table [33]) yielding products of ~1.3 kb. PCR products of the appropriate size were excised from agarose gels using a QIAquick Gel Extraction kit (QIAGEN, Germany) and used for a subsequent round of PCR amplification with the degenerate primers AMXU368F and AMXU820R (S1 Table). This primer pair is diagnostic of Planctomycetes capable of performing anammox and generates an expected product size of ~470 bp [34]. PCR products were cleaned using a QIAquick PCR Purification Kit (QIAGEN, Germany), directly ligated into the pCR4.0™-TOPO™ vector (Invitrogen, USA), and transformed into chemically competent *E*. *coli* cells (One Shot™ TOP10; Invitrogen, USA). Recombinant clones were selected on LB agar supplemented with ampicillin (50 μg/ml). Colony PCR was performed on isolated colonies by centrifuging 100 μl of turbid culture for 3 minutes at 13,000 rpm. The pellets were suspended in 100 μl Milli-Q water and then incubated for 10 minutes at 95˚C. Following centrifugation for 2 minutes at 10,000 rpm, the supernatant was transferred to a new, sterile, 0.5 ml tube and stored at -20˚C. Colony PCR was performed using M13F and M13R primers that recognize the cloning vector. Twenty-five colonies were screened for each sample type (T30 and T34 sand filters) and insert sizes of both ~470 bp and ~1.3 kb were obtained. Plasmid extractions were performed with a QIAprep® Spin Miniprep kit (QIAGEN, Germany) for all clones and sent to the University of Tennessee’s Genomics Core for Sanger sequencing using the M13F and M13R primers. Sequences were visually trimmed to exclude the plasmid backbone and ensure only high-quality sequence data was analyzed. Forward and reverse sequences from individual 470 bp and 1.3 kb insert clones were assembled using the Assemble Sequences tool in CLC Genomics Workbench. Assembled sequences were analyzed using BLASTn to retrieve the most closely related 16S rRNA gene sequences in the NCBI nr database [35].

The Map Reads to References tool in CLC Genomics Workbench was used to map 16S rRNA gene sequences from the Illumina sequencing effort for tanks T30 and T34 (water and sand filter samples) to the Planctomycete-targeted nested PCR sequences. Mapping parameters were set to record sequences with at least 97% sequence identity and a minimum coverage of 50% of the nested PCR sequences. A maximum likelihood phylogenetic tree based on the HKY85 nucleotide substitution model of 16S rRNA gene sequences was generated using PhyML 3.0 software package (http://www.atgc-montpellier.fr/phyml).

## Results

As a first step in assessing the presence, diversity, relative abundance, and temporal stability of potential anammox organisms within commercial aquaria, we performed a 16S rRNA gene survey of the microbial communities present in both surface waters and filtration systems for four exhibits within the Tennessee Aquarium. Surface water samples represent the planktonic microbial communities of the aquaria, which are well-mixed and oxygenated. Sand from the filtration systems represent sediment-like environments, within which zones of anoxia are dominant just below the surface. These exhibits, sampled weekly for four consecutive weeks, are characterized by distinct salinities (two marine, two freshwater), temperature profiles (two temperate [~25°F], two cold [~10°F and 17°F]), resident macrofauna, volumes, and turn-over rates (Table 1). Water chemistry measurements were collected in tandem with microbial community sampling to quantitatively compare any fluctuations (S2 Table).

### Microbial diversity was stable over time and distinct for each exhibit

**Fig 1:**
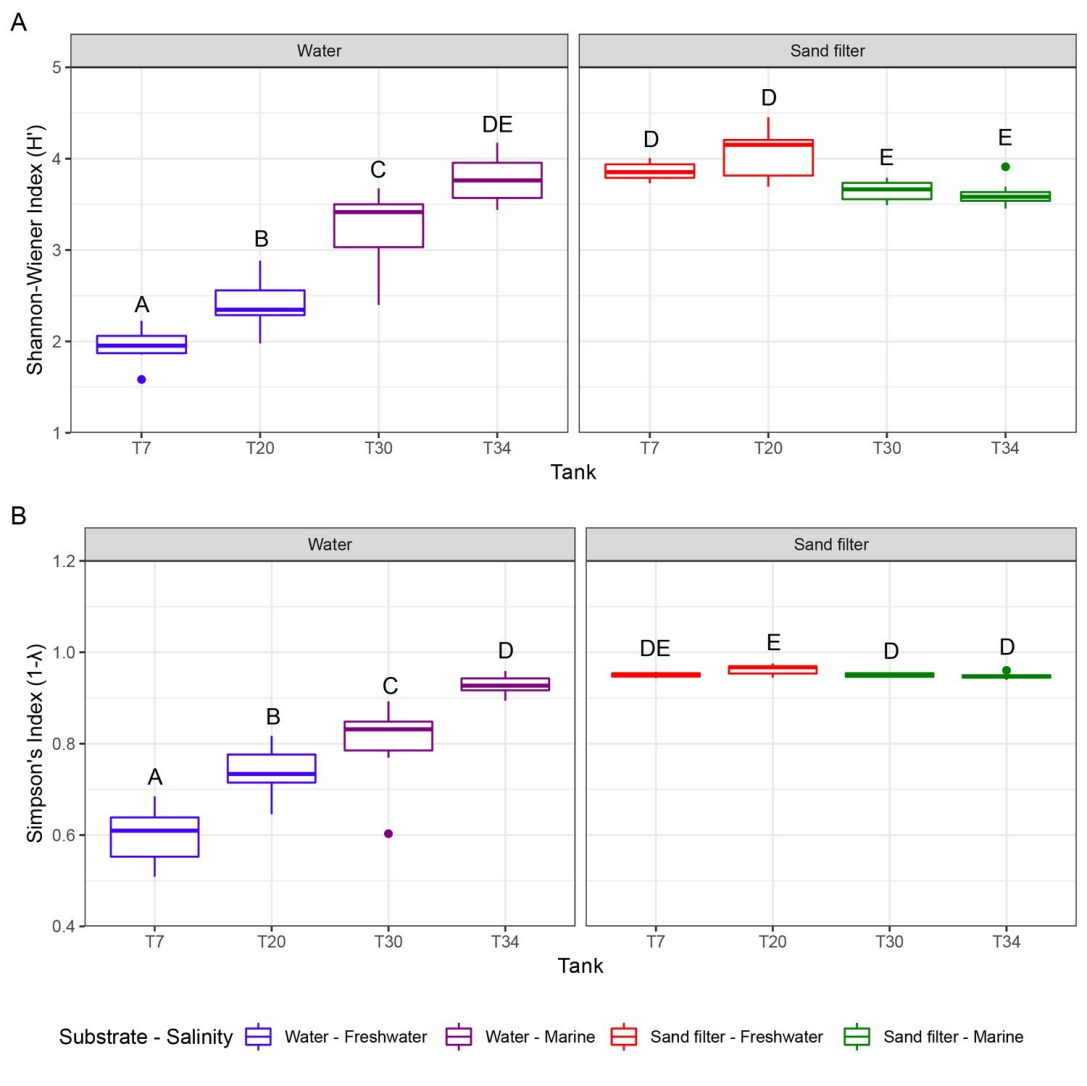
Shannon-Wiener Diversity (A) and Simpson’s Diversity (B) estimates for all Tennessee aquarium habitat microbial communities. Plots represent samples taken over the one-month data collection period with two substrate types (water and sand filter) represented per tank and depict the median (bold line), 25th and 75th percentiles (box), 1.5 times the interquartile ranges (whiskers), and outliers (dots). Letters represent significant differences (p ≤ 0.01) between samples determined by pairwise Wilcoxon tests for each diversity index. Samples labeled with different letters indicate statistically significant differences between them while samples with shared letters have no statistical difference.

A broad-based microbial diversity approach revealed the temporal stability and diversity of microbial communities within these systems. A total of 95 samples were analyzed, yielding 9,912,916 reads passing quality control, resulting in an average of 104,346 reads per sample. A total of 3,845 OTUs (clustered according to >97% identity to cultured organisms) were identified amongst all the samples. Overall, microbial community diversity, as assessed by Shannon-Weiner and Simpson indices, was stable for each habitat over the month-long sampling (Fig 1). With the exception of T34, Shannon-Wiener diversity (H’) was significantly higher for communities isolated from sand filters relative to water (Fig 1A). Marine water communities exhibited higher H’ values than their freshwater counterparts (Fig 1A). Diversity calculated using the Simpson index (1-λ) also indicated higher diversity in sand filter versus water samples (Fig 1B). The average Simpson’s index of diversity for sand filter samples was 0.95, whereas the average for water samples was 0.77.

**Fig 2:**
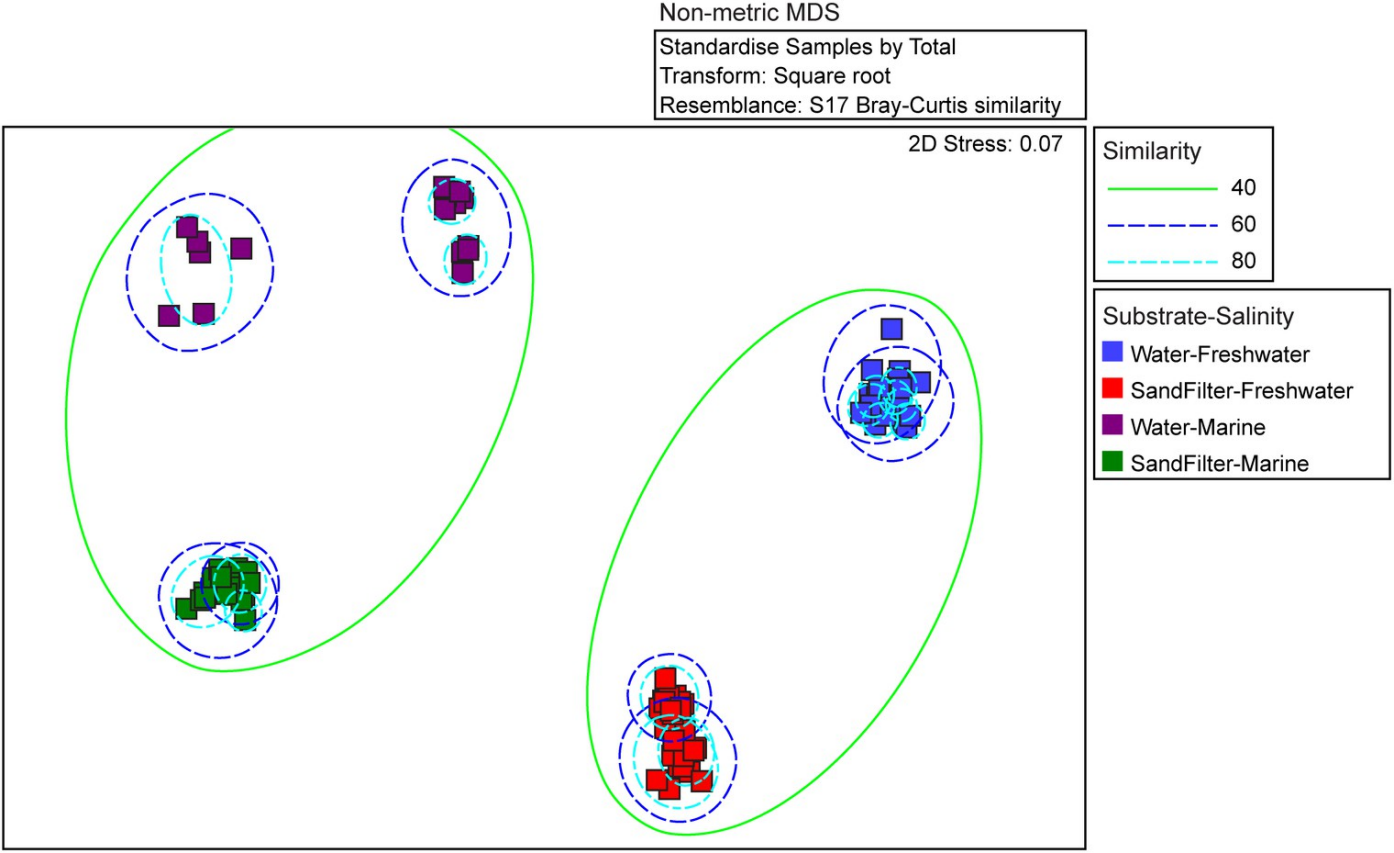
Non-metric multidimensional scaling (NMDS) analysis comparing microbial community composition from samples collected over the one-month sampling period. Samples are colored by substrate type and salinity. Each square represents a single timepoint. Ellipses represent percent similarity as calculated by the Bray-Curtis distance matrix.

Non-metric multidimensional scaling (NMDS) analysis for the one-month sampling period showed that microbial communities clustered according to habitat salinity and substrate-type (Fig 2). Habitats with differing salinity values (marine vs. freshwater) shared ≥40% similarity (Bray-Curtis metric) in their microbial community compositions. Communities present in the same substrate-type (water vs. sand filter) shared >60% similarity (Bray-Curtis metric). Unique to the marine water samples, temperature was also deterministic of community composition, with communities sharing ≥69% similarity when grouped by temperature (cold vs. temperate).

### Linear discriminant analysis revealed abundant biomarker OTUs

**Fig 3:**
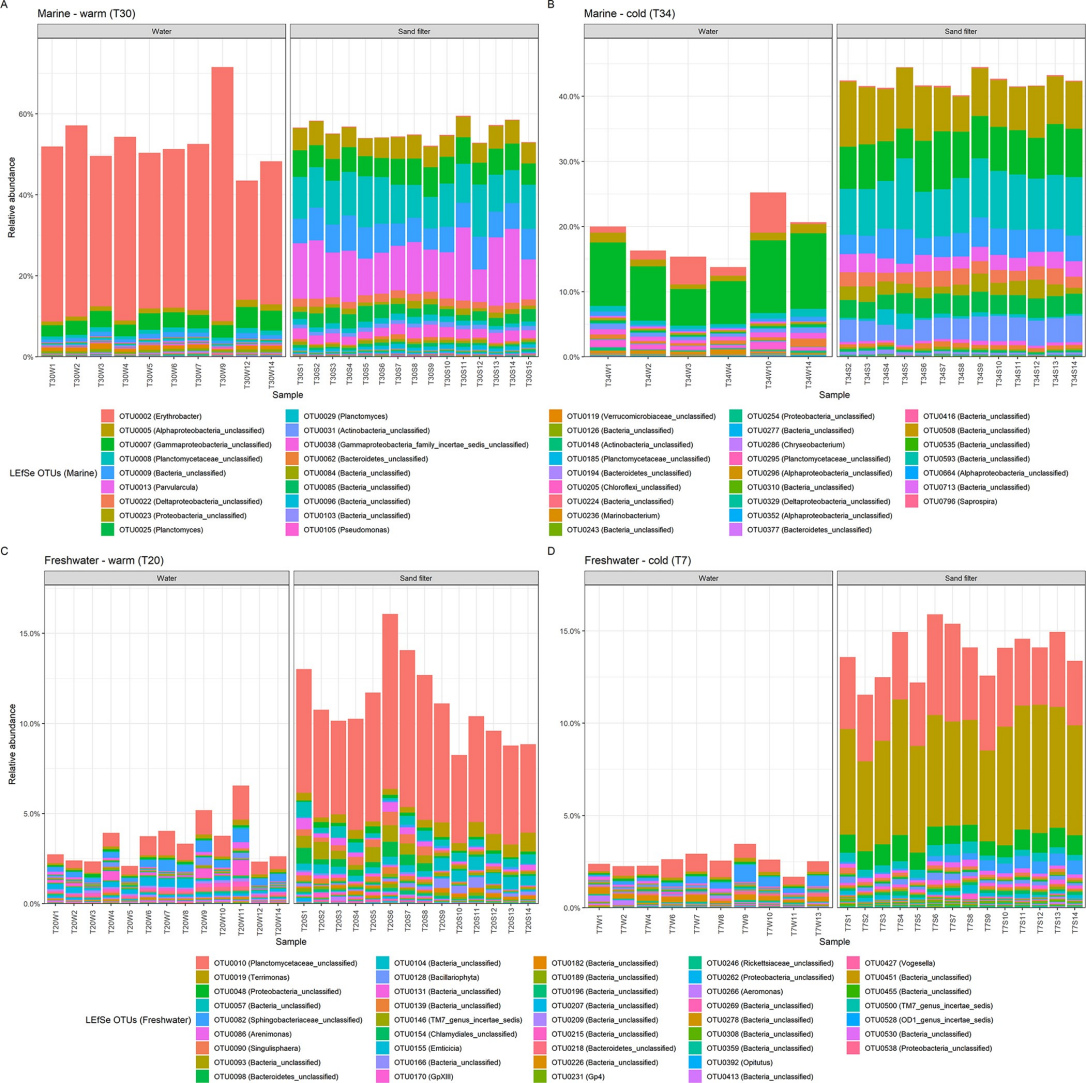
Relative abundance of biomarker OTUs in each habitat over time. Biomarker OTUs were determined by linear discriminant analysis for marine (A, B) and freshwater (C, D) tanks. See S5 Table for taxonomic identification of OTUs.

**Fig 4:**
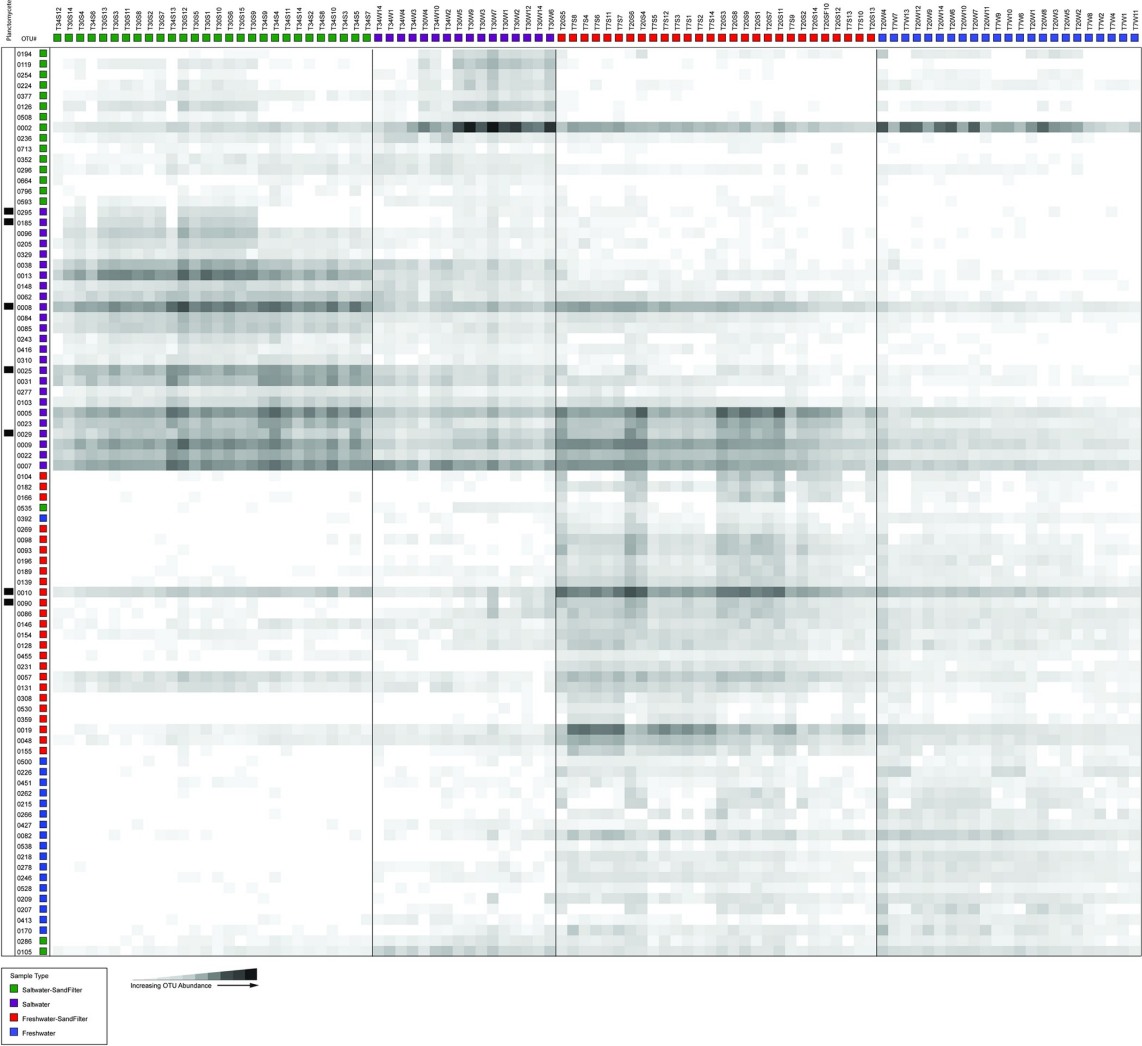
Heatmap displaying the abundance of biomarker OTUs identified from each habitat. Planctomycete OTUs are indicated by black bars left of OTU numbers. See S5 Table for taxonomic identification of OTUs.

Each habitat had unique dominant microbial OTUs as determined by linear discriminant analysis (LDA) (Fig 3). Sample biomarker OTUs determined by LDA were identified based on 16S rRNA gene abundances that characterized the differences between habitats [36]. For marine samples, the cumulative relative abundance of all biomarkers describing an individual habitat ranged from 13–73% of library reads (Fig 3A and 3B). Relative abundance of freshwater biomarkers ranged from 2–16% of library reads (Fig 3C and 3D). Higher relative abundances of biomarker OTUs were observed in sand filter samples as compared to water samples, regardless of salinity or temperature. In communities derived from the marine temperate water samples (designated T30W), a single OTU (OTU0002), identified as a member of the genus *Erythrobacter*, dominated the microbial community, comprising 43% of the reads (Fig 3A; S3 Table). Seven biomarker OTUs belonged to the order Planctomycete and were diagnostic for either marine (OTU0008, OTU0025, OTU0029, OTU0185, OTU0295) or freshwater (OTU0010 and OTU0090) sand filter habitats (Fig 4; S3 Table). One sequence type (OTU0008) comprised ~10% of the sequence reads from both the cold and temperate marine sand filtration microbial communities (T30S and T34). A second sequence type, OTU0025, comprised ~2.5% of these same communities (Fig 3A and 3B; S3 Table).

### Metagenomic analyses indicated genetic potential for complete metabolic pathways

Samples of water and sand filtration systems for each of the four habitats, as well as a denitrification system processing water from T30, were used to create metagenomic libraries. From the 9 samples, a total of 30,220,660 reads were obtained. The average contig size for these libraries ranged from ~1800–7500 bp (S6 Table), indicating these are diverse microbial communities and a greater depth of sequencing is needed. Despite the relatively low coverage, the metagenome data did indicate the genetic potential for complete pathways of assimilatory sulfate reduction, dissimilatory sulfate reduction and oxidation, and sulfur-oxidation (S2A Fig). The genetic potential for complete pathways of dissimilatory nitrate reduction, assimilatory nitrate reduction, denitrification, and nitrification were also found (S2B Fig). Regarding the anammox pathway, the precursor genes *nirK* and *nirS*, which are linked to the conversion of nitrite to nitric oxide in denitrification, were identified in all four aquarium habitats (S2B Fig). Yet, neither of the essential diagnostic genes for anammox, hydrazine synthase (*hzs*) or hydrazine dehydrogenase (*hdh*), were detected [37, 38]. Additionally, none of the 16S rRNA genes from reference cultured Planctomycetes isolates mapped to contigs. However, as anammox Planctomycetes that are functionally active often comprise <1% of their community [23, 24] and these libraries represented relatively low coverage of these microbial communities, it was reasoned that additional, targeted approaches were needed to more fully address the question of their presence and potential functionality.

### Targeted 16S rRNA gene analysis identified multiple Planctomycetes

**Fig 5:**
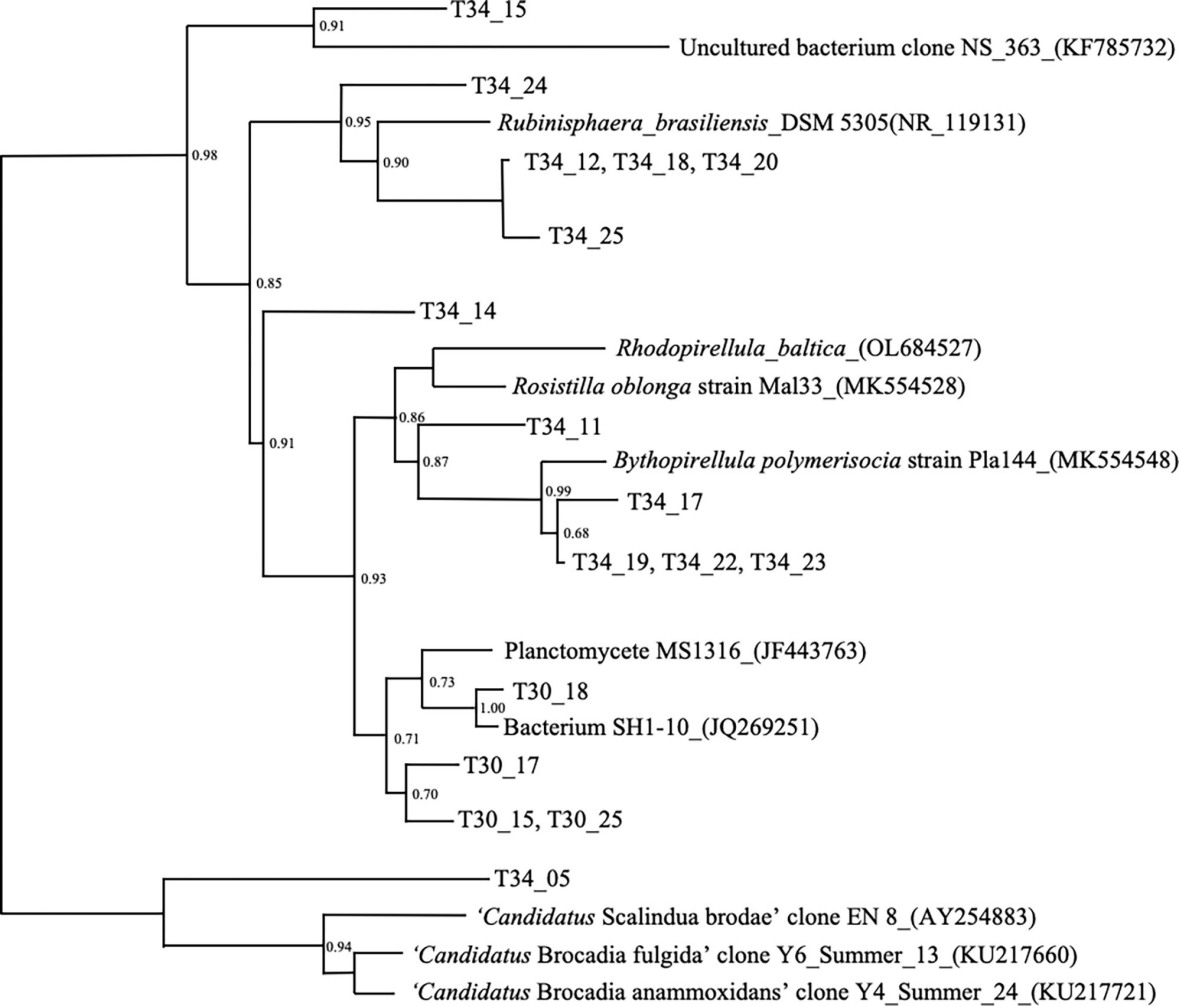
Maximum likelihood phylogenetic tree of aquarium-derived ribosomal 16S rRNA genes. Sequences were identified within marine temperate (T30) and marine cold (T34) sand filter samples. Reference sequences of bacteria belonging to the Planctomycete phylum are included with GenBank numbers provided in parentheses. Numbers at tree nodes represent bootstrap support values from 1000 replications. Only those values above 0.50 are represented. *E*. *coli* strain JNQH497 (CP091925) was used as an outgroup.

All samples were further analyzed via Planctomycete-targeted 16S rRNA gene analysis using a nested PCR approach, where the first round of PCR is intended to amplify all Planctomycete sequences and the second round is specific for the anammox-specific sublineage (S1 Fig). However, only sand filter samples from the two marine tanks (T30S and T34S) tanks yielded PCR products. These products were cloned and representatives further analyzed. An initial size survey of 50 clones revealed that <20% of the clones had the anticipated insert size of ~500 bp insert; the remaining clones had insert sizes of ~1.3 kb, which is equivalent to the product anticipated from the first round of amplification. Ten unique sequences were obtained and the closest matches to sequences in the NCBI database ranged from 88–97% sequence identity (S5 Table). The sequences derived from the longer amplification products had their closest matches to 16S rRNA genes from non-anammox Planctomycetes and analysis of these sequences revealed mismatches with the anammox-specific PCR primer sets employed in the second round of amplification (e.g., S3 Fig). Of the clones representing the size expected for anammox-lineage specific amplification, a single clone (T34-CFU-05) had homology to 16S rRNA genes, with its closest match (88% identity) to a clone denoted as *Candidatus* ‘Brocadia fulgida’ (KU217660; S5 Table). Several clones were not 16S rRNA genes, indicative of non-specific amplification. A phylogenetic tree of the clone-derived 16S rRNA sequences supports the homology searches and places T34-CFU-05 near the anammox subgroup within the Planctomycetes (Fig 5). The remaining aquarium-derived sequences from each of the two marine tanks are found on separate branches of the tree.

## Discussion

Commercial, large-scale aquaria are engineered to achieve high water clarity, efficient removal of toxic chemical species, and stable environmental conditions to support the resident macrofaunal species. The contributions of resident microorganisms to support a healthy aquatic environment, principally through the transformation of toxic nitrogen species via nitrification and denitrification, has been recognized for over a hundred years [39, 40]. However, anammox, a process increasingly employed in wastewater treatment, represents an alternative microbial pathway for processing the most common toxic nitrogen species in aquaria (i.e., ammonium and nitrite), and one that has not been widely considered in this context [41, 42]. Anammox Planctomycetes are frequently present in wastewater treatment plants, freshwater lakes, marine suboxic zones, and coastal sediments [20]. The low abundance (typically <1%) of anammox bacteria in natural systems initially prevented identification of these microorganisms. However, the broader understanding of their significant contributions to nitrogen cycling is now well understood and evidence to date suggests group members are present in most, if not all, aquatic systems [20, 22–24]. In order to better understand the potential of anammox as a viable process for N control in commercial aquaria, we applied a series of culture-independent approaches to understand the microbial community structure and potential metabolic functions within Tennessee Aquarium exhibits that differ in chemical and physical properties.

Our broad-based microbial community survey identified Planctomycetes phylum representatives in all aquaria samples. Seven Planctomycete OTUs were identified as diagnostic biomarkers for the different habitats and their relative abundances varied across the microbial communities sampled. These organisms have the greatest representation in both the temperate and cold marine sand filtration systems, where they represent >10% of the microbial communities. Planctomycetes known to carry out anammox appear to be restricted to specific lineages within the phylum and can be best identified by examining variable regions 2 and 3 (V2/V3) of the 16S rRNA gene [43]. Our microbiome analysis utilized the Earth Microbiome primers, which target the V4 region of the 16S rRNA gene. Thus, this approach is not immediately diagnostic of anammox-lineage Planctomycetes. However, it does provide insight into the relative abundance of members of this phylum as well as the full composition and stability of the microbial communities in these systems, all of which are valuable for cross-system comparisons. These types of comparisons will ultimately prove useful in elucidating the role that aquarium microbiomes play in maintaining healthy water chemistry and macrofaunal communities.

In assessing the Tennessee Aquarium metagenomic datasets for genes diagnostic of anammox (i.e., *hdh* and *hzs*), neither were identified by sequence homology to functionally confirmed genes. However, two potential precursor proteins (encoded by *nirK* and *nirS*) were identified. These proteins encode nitrite reductases that produce ammonia, a key substrate for anammox, but also denitrification [40]. These genes were previously identified in the metagenomes derived from the Georgia Aquarium’s *Ocean Voyager* tank (S2 Fig [17, 37, 38]). The Georgia Aquarium study assessed the microbial communities residing with sulfur-driven denitrification (SDN) reactors with a focus on anaerobic denitrification performed by sulfur-oxidizing bacteria as a method to offload toxic nitrogen species [17]. The SDN systems contained highly diverse microbial communities, and steps of the SDN pathway were predicted to be partitioned amongst community members [17]. All genes involved in nitrogen and sulfur metabolisms identified in Georgia Aquarium *Ocean Voyager* communities were also found in the Tennessee Aquarium marine metagenomes. Furthermore, the Tennessee Aquarium samples contained additional evidence for nitrogen and sulfur metabolism not evident in the *Ocean Voyager* metagenomes (S2 Fig; [17]). As was observed in the *Ocean Voyager* tank [17], the suite of genes required for individual pathways may be distributed across several organisms in the Tennessee Aquarium communities. A more in-depth analysis of the genomic content and phylogenetic context of functional genes is necessary to robustly address this question.

Considering both the absence of functional anammox diagnostic genes and the relatively low sequence coverage of our metagenomes, a 16S rRNA PCR amplification approach specifically targeting anammox Planctomycetes was ultimately employed. This approach provided additional evidence for the presence of Planctomycete phylum representatives within the marine (T30 and T34) sand filter samples (Fig 5). Whether these sequences represent organisms capable of anammox remains unclear. None of the cloned sequences shared >97% identity to any sequences in public databases, and many shared <95% identity (S6 Table). One clone sequence (T34-CFU-5) showed little homology to sequences in the NCBI database, with greatest homology to a candidate anammox lineage member at 88% identity (Fig 5; S4 Table). This low sequence identity is in itself intriguing and further work isolating strains or performing genome assemblies will be needed to characterize this potentially novel bacterium.

The lack of conclusive data supporting the presence of anammox planctomycetes within the Tennessee Aquarium tanks does not necessarily indicate that these bacteria are absent in these environments, nor does it rule out the possibility of bioaugmentation or biostimulation to promote the anammox reaction in these systems. The presence of complete pathways for denitrification and nitrification indicates potential for these habitats to sustain levels of nitrite and ammonium that would enable the anammox reaction [22]. Additionally, wastewater treatment facilities represent systems that contain high concentrations of nitrate, nitrite, and ammonium, yet anammox bacteria are not typically naturally present [44, 45]. Instead, they are added to these systems, typically within a bioreactor that provides sufficiently low reduction potential to support the growth of these bacteria [46–49]. Indeed, Tal et al. (2006), reported successful anammox activity in fixed film biofilters within marine aquaculture systems [49]. These biofilters are structurally and functionally similar to the anammox bioreactors used in wastewater treatment plants [45–49] and represent a path forward for application of analogous systems in commercial aquaria.

## Supporting information

S1 Table PCR Primers used throughout this study. (DOCX)

S2 Table Metadata for each tank over time. (DOCX)

S3 Table Taxonomic identity and average relative abundance of select OTUs from linear discriminant analysis. (DOCX)

S4 Table List of sequenced clones and their closest homologs in NCBI database. (DOCX)

S5 Table Library reads for each sample and taxonomic affiliations for all OTUs. (XLSX)

S6 Table Metagenomic library statistics. (XLSX)

S1 Fig Diagram of expected product sizes from nested PCR. The first round of PCR selectively amplifies 16S rRNA genes within the phylum Planctomycete [32, 33]. The second round of PCR only amplifies those Planctomycete 16S rRNA genes that belong to the anammox subgroup [34].
(DOCX)

S2 Fig KEGG generated sulfur (A) and nitrogen (B) transformation pathways. The genes identified in the Tennessee Aquarium metagenomic survey are highlighted in green. Genes identified in the Ocean Voyager tank at the Georgia Aquarium [12] are outlined in orange.
(DOCX)

S3 Fig Diagram showing potential binding sites of primers used in the Planctomycete-targeted nested PCR on a 1.3 kb assembled sequence (T30-CFU-12). The Primer Mapping tool in CLC Genomics Workbench was utilized to map all four primers from the nested PCR onto the assembled 1.3 kb sequence. Primer mapping parameters were set to allow primers to bind to the sequence with a maximum of 10 mismatches and at least 80% coverage.
(DOCX)

## References

[1] Lynch JB, Hsiao EY. Microbiomes as sources of emergent host phenotypes. Science. 2019;365(6460):1405–8. doi: 10.1126/science.aay0240 31604267

[2] Teng Y, Chen W. Soil Microbiomes-a Promising Strategy for Contaminated Soil Remediation: A Review. Pedosphere. 2019;29(3):283–97.

[3] De Vrieze J, Boon N, Verstraete W. Taking the technical microbiome into the next decade. Environ Microbiol. 2018;20(6):1991–2000. doi: 10.1111/1462-2920.14269 29745026

[4] Zhu YG, Zhao Y, Zhu D, Gillings M, Penuelas J, Ok YS, et al. Soil biota, antimicrobial resistance and planetary health. Environ Int. 2019;131:7. doi: 10.1016/j.envint.2019.105059 31374443

[5] Grossart HP, Massana R, McMahon KD, Walsh DA. Linking metagenomics to aquatic microbial ecology and biogeochemical cycles. Limnol Oceanogr. 2020;65:S2–S20.

[6] Proctor L, LoTempio J., Marquitz A. et al. A review of 10 years of human microbiome research activities at the US National Institutes of Health, Fiscal Years 2007–2016. Microbiome. 2019;7(31). 10.1186/s40168-019-0620-y PMC6391833 30808411

[7] Zorz J, Willis C, Comeau AM, Langille MGI, Johnson CL, Li WKW, et al. Drivers of Regional Bacterial Community Structure and Diversity in the Northwest Atlantic Ocean. Frontiers in Microbiology. 2019;10(281). doi: 10.3389/fmicb.2019.00281 30846975 PMC6393369

[8] Paerl HW, Dyble J, Twomey L, Pinckney JL, Nelson J, Kerkhof L. Characterizing man-made and natural modifications of microbial diversity and activity in coastal ecosystems. Antonie Van Leeuwenhoek. 2002;81(1–4):487–507. doi: 10.1023/a:1020561422706 12448745

[9] Damashek J, Francis CA. Microbial Nitrogen Cycling in Estuaries: From Genes to Ecosystem Processes. Estuaries Coasts. 2018;41(3):626–60.

[10] Wilmes P, Bond PL. Microbial community proteomics: elucidating the catalysts and metabolic mechanisms that drive the Earth’s biogeochemical cycles. Curr Opin Microbiol. 2009;12(3):310–7. doi: 10.1016/j.mib.2009.03.004 19414280

[11] Van Bonn W, LaPointe A, Gibbons SM, Frazier A, Hampton-Marcell J, Gilbert J. Aquarium microbiome response to ninety-percent system water change: Clues to microbiome management. Zoo Biol. 2015;34(4):360–7. doi: 10.1002/zoo.21220 26031788 PMC4852745

[12] Patin NV, Pratte ZA, Regensburger M, Hall E, Gilde K, Dove ADM, et al. Microbiome Dynamics in a Large Artificial Seawater Aquarium. Appl Environ Microbiol. 2018;84(10). 10.1128/AEM.00179-18 PMC5930379 29523545

[13] Ip YK, Chew SF. Ammonium production, excretion, toxicity, and defense in fish: a review. Front Physiol. 2010;1:20. doi: 10.3389/fphys.2010.00020 21423375 PMC3059970

[14] Bellucci M, Curtis TP. Ammonium-oxidizing bacteria in wastewater. In: Klotz MG, Stein LY, editors. Methods in Enzymology, Vol 46: Research on Nitrification and Related Processes, Pt B. Methods in Enzymology. 496. San Diego: Elsevier Academic Press Inc; 2011. p. 269–86. 10.1016/B978-0-12-386489-5.00011-7 21514468

[15] Abeliovich A. The Nitrite-Oxidizing Bacteria. Dworkin M, Falkow S, Rosenberg E, Schleifer KH, Stackebrandt E, editors. New York: Springer; 2006. 861–72 p.

[16] Kim JH, Kang YJ, Kim KI, Kim SK, Kim JH. Toxic effects of nitrogenous compounds (ammonium, nitrite, and nitrate) on acute toxicity and antioxidant responses of juvenile olive flounder, *Paralichthys olivaceus*. Environ Toxicol Pharmacol. 2019;67:73–8. doi: 10.1016/j.etap.2019.02.001 30763818

[17] Burns AS, Padilla CC, Pratte ZA, Gilde K, Regensburger M, Hall E, et al. Broad Phylogenetic Diversity Associated with Nitrogen Loss through Sulfur Oxidation in a Large Public Marine Aquarium. Appl Environ Microbiol. 2018;84(20). 10.1128/AEM.01250-18 PMC6182901 30097447

[18] Shi XF, Tal G, Hankins NP, Gitis V. Fouling and cleaning of ultrafiltration membranes: A review. J Water Process Eng. 2014;1:121–38.

[19] Meng FG, Zhang SQ, Oh Y, Zhou ZB, Shin HS, Chae SR. Fouling in membrane bioreactors: An updated review. Water Res. 2017;114:151–80. doi: 10.1016/j.watres.2017.02.006 28237783

[20] van Niftrik L, Jetten MSM. Anaerobic Ammonium-Oxidizing Bacteria: Unique Microorganisms with Exceptional Properties. Microbiol Mol Biol Rev. 2012;76(3):585 doi: 10.1128/MMBR.05025-11 22933561 PMC3429623

[21] Jetten MSM, van Niftrik L, Strous M, Kartal B, Keltjens JT, Op den Camp HJM. Biochemistry and molecular biology of anammox bacteria. Crit Rev Biochem Mol Biol. 2009;44(2–3):65–84. doi: 10.1080/10409230902722783 19247843

[22] Kartal B, de Almeida NM, Maalcke WJ, Op den Camp HJM, Jetten MSM, Keltjens JT. How to make a living from anaerobic ammonium oxidation. FEMS Microbiology Reviews. 2013;37(3):428–61. doi: 10.1111/1574-6976.12014 23210799

[23] Arrigo KR. Marine microorganisms and global nutrient cycles. Nature. 2005;437(7057):349–55. doi: 10.1038/nature04159 16163345

[24] Lam P, Kuypers MMM. Microbial Nitrogen Cycling Processes in Oxygen Minimum Zones. In: Carlson CA, Giovannoni SJ, editors. Annual Review of Marine Science, Vol 3. Annual Review of Marine Science. 3. Palo Alto: Annual Reviews; 2011. p. 317–45. doi: 10.1146/annurev-marine-120709-142814 21329208

[25] Ma B, Wang SY, Cao SB, Miao YY, Jia FX, Du R, et al. Biological nitrogen removal from sewage via anammox: Recent advances. Bioresour Technol. 2016;200:981–90. doi: 10.1016/j.biortech.2015.10.074 26586538

[26] Lackner S, Gilbert EM, Vlaeminck SE, Joss A, Horn H, van Loosdrecht MCM. Full-scale partial nitritation/anammox experiences—An application survey. Water Res. 2014;55:292–303. doi: 10.1016/j.watres.2014.02.032 24631878

[27] Thompson LR, Sanders JG, McDonald D, Amir A, Ladau J, Locey KJ, et al. A communal catalogue reveals Earth’s multiscale microbial diversity. Nature. 2017;551(7681):457–63. doi: 10.1038/nature24621 29088705 PMC6192678

[28] Schloss PD, Westcott SL, Ryabin T, Hall JR, Hartmann M, Hollister EB, et al. Introducing mothur: Open-Source, Platform-Independent, Community-Supported Software for Describing and Comparing Microbial Communities. Appl Environ Microbiol. 2009;75(23):7537–41. doi: 10.1128/AEM.01541-09 19801464 PMC2786419

[29] Afgan E, Baker D, Batut B, van den Beek M, Bouvier D, Čech M, et al. The Galaxy platform for accessible, reproducible and collaborative biomedical analyses: 2018 update. Nucleic Acids Research. 2018;46(W1):W537–W44. doi: 10.1093/nar/gky379 29790989 PMC6030816

[30] Kanehisa M, Sato Y, Morishima K. BlastKOALA and GhostKOALA: KEGG Tools for Functional Characterization of Genome and Metagenome Sequences. J Mol Biol. 2016;428(4):726–31. doi: 10.1016/j.jmb.2015.11.006 26585406

[31] Kanehisa M, Goto S. KEGG: Kyoto Encyclopedia of Genes and Genomes. Nucleic Acids Research. 2000;28(1):27–30. doi: 10.1093/nar/28.1.27 10592173 PMC102409

[32] Neef A, Amann R, Schlesner H, Schleifer KH. Monitoring a widespread bacterial group: in situ detection of planctomycetes with 16S rRNA-targeted probes. Microbiology. 1998;144 (Pt 12):3257–66. doi: 10.1099/00221287-144-12-3257 9884217

[33] Zheng DD, Alm EW, Stahl DA, Raskin L. Characterization of universal small-subunit rRNA hybridization probes for quantitative molecular microbial ecology studies. Appl Environ Microbiol. 1996;62(12):4504–13. doi: 10.1128/aem.62.12.4504-4513.1996 8953722 PMC168277

[34] Amano T, Yoshinaga I, Okada K, Yamagishi T, Ueda S, Obuchi A, et al. Detection of anammox activity and diversity of anammox bacteria-related 16S rRNA genes in coastal marine sediment in japan. Microbes Environ. 2007;22(3):232–42.

[35] Altschul SF, Gish W, Miller W, Myers EW, Lipman DJ. Basic local alignment search tool. J Mol Biol. 1990;215(3):403–10. doi: 10.1016/S0022-2836(05)80360-2 2231712

[36] Segata N, Izard J, Waldron L, Gevers D, Miropolsky L, Garrett WS, et al. Metagenomic biomarker discovery and explanation. Genome Biol. 2011;12(6):18. doi: 10.1186/gb-2011-12-6-r60 21702898 PMC3218848

[37] Hirsch MD, Long ZT, Song B. Anammox Bacterial Diversity in Various Aquatic Ecosystems Based on the Detection of Hydrazine Oxidase Genes (*hzoA/hzoB*). Microb Ecol. 2011;61(2):264–76. doi: 10.1007/s00248-010-9743-1 20838786

[38] Harhangi HR, Le Roy M, van Alen T, Hu BL, Groen J, Kartal B, et al. Hydrazine Synthase, a Unique Phylomarker with Which to Study the Presence and Biodiversity of Anammox Bacteria. Appl Environ Microbiol. 2012;78(3):752–8. doi: 10.1128/AEM.07113-11 22138989 PMC3264106

[39] Cooper EA. Denitrification as a means of sewage purification. Biochem J. 1921;15(4):513–5. doi: 10.1042/bj0150513 16743019 PMC1259012

[40] Pajares S, Ramos R. Processes and Microorganisms Involved in the Marine Nitrogen Cycle: Knowledge and Gaps. Front Mar Sci. 2019;6:33.

[41] Gonzalez-Martinez A, Munoz-Palazon B, Rodriguez-Sanchez A, Gonzalez-Lopez J. New concepts in anammox processes for wastewater nitrogen removal: recent advances and future prospects. FEMS Microbiol Lett. 2018;365(6):10. doi: 10.1093/femsle/fny031 29438563

[42] Vandegraaf AA, Mulder A, Debruijn P, Jetten MSM, Robertson LA, Kuenen JG. Anaerobic oxidation of ammonium is a biologically mediated process. Appl Environ Microbiol. 1995;61(4):1246–51. doi: 10.1128/aem.61.4.1246-1251.1995 7747947 PMC167380

[43] Schmid M, Schmitz-Esser S, Jetten M, Wagner M. 16S-23S rDNA intergenic spacer and 23S rDNA of anaerobic ammonium-oxidizing bacteria: implications for phylogeny and in situ detection. Environ Microbiol. 2001;3(7):450–9. doi: 10.1046/j.1462-2920.2001.00211.x 11553235

[44] Ganesan S, Vadivelu VM. Effect of storage conditions on maintaining anammox cell viability during starvation and recovery. Bioresour Technol. 2020;296:9. doi: 10.1016/j.biortech.2019.122341 31711905

[45] Chen WJ, Chen SD, Hu F, Liu WR, Yang DH, Wu J. A novel anammox reactor with a nitrogen gas circulation: performance, granule size, activity, and microbial community. Environ Sci Pollut Res.11. 10.1007/s11356-020-08432-w 32198688

[46] van der Star WRL, Miclea AI, van Dongen U, Muyzer G, Picioreanu C, van Loosdrecht MCM. The membrane bioreactor: A novel tool to grow anammox bacteria as free cells. Biotechnol Bioeng. 2008;101(2):286–94. doi: 10.1002/bit.21891 18421799

[47] Oshiki M, Awata T, Kindaichi T, Satoh H, Okabe S. Cultivation of Planktonic Anaerobic Ammonium Oxidation (Anammox) Bacteria Using Membrane Bioreactor. Microbes Environ. 2013;28(4):436–43. doi: 10.1264/jsme2.me13077 24200833 PMC4070702

[48] Wu N, Zeng M, Zhu BF, Zhang WY, Liu HX, Yang L, et al. Impacts of different morphologies of anammox bacteria on nitrogen removal performance of a hybrid bioreactor: Suspended sludge, biofilm and gel beads. Chemosphere. 2018;208:460–8. doi: 10.1016/j.chemosphere.2018.06.012 29886334

[49] Tal Y, Watts JEM, Schreier HJ. Anaerobic ammonium-oxidizing (anammox) bacteria and associated activity in fixed-film biofilters of a marine recirculating aquaculture system. Appl Environ Microbiol. 2006;72(4):2896–904. doi: 10.1128/AEM.72.4.2896-2904.2006 16597996 PMC1448996

