# Correction: Microbiomes and Planctomycete diversity in large-scale aquaria habitats

**DOI:** 10.1371/journal.pone.0307131

**Published:** 2024-07-11

**Authors:** Claire E. Elbon, Gary R. LeCleir, Matthew J. Tuttle, Sophie K. Jurgensen, Thomas G. Demas, Christian J. Keller, Tina Stewart, Alison Buchan

There are errors in the Funding statement. The correct Funding statement is as follows: This study was supported by the National Science Foundation in the form of funds to AB [OCE-1737237] and GRL [DBI-2050743]. This study was also supported by the Tennessee Aquarium, Inc. in the form of funds to AB.
