# Supplementary material for: Microbiomes and Planctomycete diversity in large-scale aquaria habitats
Source: PLoS One. 2022 May 12;17(5):e0267881. doi: 10.1371/journal.pone.0267881 (PMC9098025; doi:10.1371/journal.pone.0267881)
Supplement: S1 Table — (DOCX) [file pone.0267881.s001.docx]

**S1 Table. PCR primers used throughout this study.**

| **Primer name** | **5’ to 3’ Sequence** | **Target** | **Reference** |
| --- | --- | --- | --- |
| 515F-Adapt | TCGTCGGCAGCGTCAGATGTGTATAAGAGACAGGTGYCAGCMGCCGCGGTAA | Bacteria and Archaea | 27 |
| 806R-Adapt | GTCTCGTGGGCTCGGAGATGTGTATAAGAGACAGGGACTACNVGGGTWTCTAAT | Bacteria and Archaea | 27 |
| Pla46F | GACCTTGCATGCCTAATCC | Planctomycetes | 32 |
| 1390R | GACGGGCGGTGTGTACAA | Bacteria | 33 |
| AMXU368F | TTCGCAATGCCCGMAAGG | Anammox Planctomycetes | 34 |
| AMXU820R | CCCTCTACYKAGTGCCC | Anammox Planctomycetes | 34 |
