# Supplementary material for: Microbiomes and Planctomycete diversity in large-scale aquaria habitats
Source: PLoS One. 2022 May 12;17(5):e0267881. doi: 10.1371/journal.pone.0267881 (PMC9098025; doi:10.1371/journal.pone.0267881)
Supplement: S2 Table — (DOCX) [file pone.0267881.s002.docx]

**S2 Table**. **Metadata for each tank over time.**

| **Tank** | **Sample Week** | **pH** | **Ammonia (ppm)** | **Nitrite (ppm)** | **Salinity (ppt)** | **Temperature range (˚F)^a^** |
| --- | --- | --- | --- | --- | --- | --- |
| T07 | 1 | 7.40 | 0.00 | 0.002 | 0.00 | 61.2 - 62.2 |
|  | 2 | n.d.^b^ | n.d. | n.d. | n.d. |  |
|  | 3 | 7.34 | 0.013 | 0.006 | 0.00 |  |
|  | 4 | 7.36 | 0.00 | 0.003 | 0.00 |  |
| T20 | 1 | 7.84 | 0.00 | 0.002 | 0.00 | 78.0 – 79.0 |
|  | 2 | n.d. | n.d. | n.d. | n.d. |  |
|  | 3 | 8.02 | 0.00 | 0.003 | 0.00 |  |
|  | 4 | 7.86 | 0.00 | 0.003 | 0.00 |  |
| T30 | 1 | 7.98 | 0.012 | 0.012 | 30.0 | 76.5 – 77.0 |
|  | 2 | 7.92 | 0.011 | 0.014 | 30.0 |  |
|  | 3 | 7.96 | 0.011 | 0.013 | 30.0 |  |
|  | 4 | 7.94 | 0.00 | 0.013 | 30.0 |  |
| T34 | 1 | 7.95 | 0.00 | 0.006 | 34.0 | 48.0 – 50.0 |
|  | 2 | 7.92 | 0.014 | 0.009 | 34.0 |  |
|  | 3 | 8.06 | 0.010 | 0.008 | 33.0 |  |
|  | 4 | 8.1 | 0.00 | 0.008 | 34.0 |  |

^a^ Temperature ranges account for the entire 1-month sampling period.

^b^ No data available.
