# Supplementary material for: Microbiomes and Planctomycete diversity in large-scale aquaria habitats
Source: PLoS One. 2022 May 12;17(5):e0267881. doi: 10.1371/journal.pone.0267881 (PMC9098025; doi:10.1371/journal.pone.0267881)
Supplement: S3 Table — (DOCX) [file pone.0267881.s003.docx]

**S3 Table**. **Taxonomic identity and average relative abundance of select OTUs from Linear Discriminant Analysis for each sample type.**

| **OTU#** | **Taxonomy** | **Average relative abundance** | | | | | | | |
| --- | --- | --- | --- | --- | --- | --- | --- | --- | --- |
|  |  | **Water** | | | | **Sand filter** | | | |
|  |  | **T07** | **T20** | **T30** | **T34** | **T07** | **T20** | **T30** | **T34** |
| 0002 | Proteobacteria Alphaproteobacteria  Sphingomonadales Erythrobacteraceae Erythrobacter | 5.85% | 16.0% | 43.4% | 2.32% | 1.57% | 0.63% | 0.16% | 0.17% |
| 0007 | Proteobacteria Gammaproteobacteria Gammaproteobacteria_unclassified | 0.56% | 0.58% | 3.59% | 8.85% | 2.93% | 1.95% | 5.75% | 6.72% |
| 0008 | Planctomycetes Planctomycetacia Planctomycetales Planctomycetaceae | 0.11% | 0.08% | 0.97% | 0.75% | 1.73% | 0.93% | 11.0% | 8.38% |
| 0010 | Planctomycetes Planctomycetacia Planctomycetales Planctomycetaceae | 0.73% | 0.88% | 0.17% | >0.01% | 4.08% | 6.91% | 0.36% | 0.43% |
| 0025 | Planctomycetes Planctomycetacia Planctomycetales Planctomycetaceae Planctomyces | >0.01% | >0.01% | 0.13% | 0.29% | >0.01% | 0.05% | 2.63% | 2.90% |
| 0029 | Planctomycetes Planctomycetacia Planctomycetales Planctomycetaceae Planctomyces | 0.08% | 0.15% | 0.40% | 0.12% | 0.31% | 1.27% | 1.26% | 0.92% |
| 0090 | Planctomycetes Planctomycetacia Planctomycetales Planctomycetaceae Singulisphaera | >0.01% | 0.02% | 0.11% | >0.01% | 0.17% | 0.37% | >0.01% | >0.01% |
| 0185 | Planctomycetes Planctomycetacia Planctomycetales Planctomycetaceae | >0.01% | >0.01% | 0.01% | >0.01% | >0.01% | >0.01% | 0.32% | >0.01% |
| 0295 | Planctomycetes Planctomycetacia Planctomycetales Planctomycetaceae | >0.01% | >0.01% | 0.02% | >0.01% | >0.01% | >0.01% | 0.11% | >0.01% |
