# Supplementary material for: Microbiomes and Planctomycete diversity in large-scale aquaria habitats
Source: PLoS One. 2022 May 12;17(5):e0267881. doi: 10.1371/journal.pone.0267881 (PMC9098025; doi:10.1371/journal.pone.0267881)
Supplement: S4 Table — (DOCX) [file pone.0267881.s004.docx]

**S4 Table**. **List of sequenced clones and their closest taxonomic hit within NCBI’s BLASTn nr database.**

| **Tank and clone number** | **Closest Taxonomic Hit** | **Accession** | **Percent Identity** |
| --- | --- | --- | --- |
| T30-CFU-15 | Planctomycete MS1316 16S ribosomal RNA gene, partial sequence | JF443763.1 | 92% |
| T30-CFU-17 | Planctomycete MS1316 16S ribosomal RNA gene, partial sequence | JF443763.1 | 92% |
| T30-CFU-18 | Bacterium SH1-10 16S ribosomal RNA gene, partial sequence | JQ269251.1 | 97% |
| T30-CFU-19 | Planctomycetes bacterium strain Pla144 16S ribosomal RNA gene, partial sequence | MK554548.1 | 95% |
| T30-CFU-22 | Planctomycetes bacterium strain Pla144 16S ribosomal RNA gene, partial sequence | MK554548.1 | 95% |
| T30-CFU-23 | Planctomycetes bacterium strain Pla144 16S ribosomal RNA gene, partial sequence | MK554548.2 | 96% |
| T30-CFU-25 | Planctomycete MS1316 16S ribosomal RNA gene, partial sequence | JQ269251.1 | 94% |
| T34-CFU-5 | Candidatus Brocadia fulgida clone Y6_Summer_13 16S ribosomal RNA gene, partial sequence | KU217660.1 | 88% |
| T34-CFU-11 | Planctomycetes bacterium strain Mal33 16S ribosomal RNA gene, partial sequence | MK554528.1 | 92% |
| T34-CFU-12 | Rubinisphaera brasiliensis DSM 5305 partial 16S rRNA gene | LR595959.1 | 96% |
| T34-CFU-14 | Planctomycetaceae bacterium D2 gene for 16S ribosomal RNA, partial sequence | LC075346.1 | 90% |
| T34-CFU-15 | Planctomycetes bacterium SCGC AAA206-C13 16S ribosomal RNA gene, partial sequence | JF488132.1 | 90% |
| T34-CFU-17 | Planctomycetes bacterium strain Pla144 16S ribosomal RNA gene, partial sequence | MK554548.1 | 93% |
| T34-CFU-18 | Rubinisphaera brasiliensis DSM 5305 partial 16S rRNA gene | LR595959.1 | 97% |
| T34-CFU-20 | Rubinisphaera brasiliensis DSM 5305 partial 16S rRNA gene | LR595959.2 | 97% |
| T34-CFU-24 | Rubinisphaera brasiliensis DSM 5305 16S ribosomal RNA, partial sequence | LR595959.2 | 89% |
| T34-CFU-25 | Rubinisphaera brasiliensis DSM 5305 partial 16S rRNA gene | LR595959.1 | 96% |
