# Supplementary material for: Microbiomes and Planctomycete diversity in large-scale aquaria habitats
Source: PLoS One. 2022 May 12;17(5):e0267881. doi: 10.1371/journal.pone.0267881 (PMC9098025; doi:10.1371/journal.pone.0267881)
Supplement: S1 Fig — The first round of PCR selectively amplifies 16S rRNA genes within the phylum Planctomycete [32, 33]. The second round of PCR only amplifies those Planctomycete 16S rRNA genes that belong to the anammox subgroup [34]. (DOCX) [file pone.0267881.s007.docx]

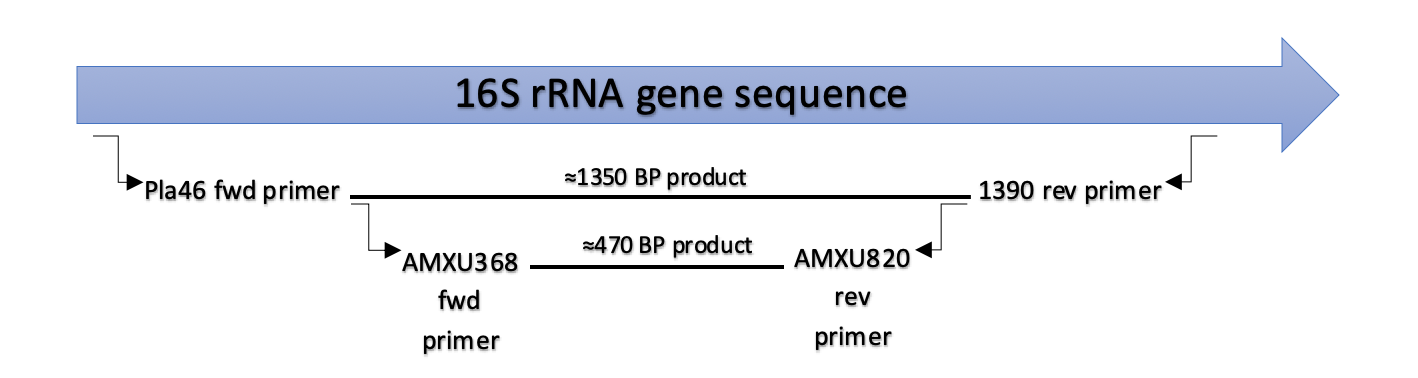


**S1 Figure**. **Diagram of expected product sizes from nested PCR**. The first round of PCR selectively amplifies 16S rRNA genes within the phylum Planctomycete (32, 33). The second round of PCR only amplifies those Planctomycete 16S rRNA genes that belong to the anammox subgroup (34).
