# Supplementary material for: Microbiomes and Planctomycete diversity in large-scale aquaria habitats
Source: PLoS One. 2022 May 12;17(5):e0267881. doi: 10.1371/journal.pone.0267881 (PMC9098025; doi:10.1371/journal.pone.0267881)
Supplement: S2 Fig — KEGG generated sulfur (A) and nitrogen (B) transformation pathways. The genes identified in the Tennessee Aquarium metagenomic survey are highlighted in green. Genes identified in the Ocean Voyager tank at the Georgia Aquarium [12] are outlined in orange. (DOCX) [file pone.0267881.s008.docx]

*__*

A

B

**S2 Figure**. **KEGG generated sulfur (A) and nitrogen (B) transformation pathways**. The genes identified in the Tennessee Aquarium metagenomic survey are highlighted in green. Genes identified in the Ocean Voyager tank at the Georgia Aquarium (12) are outlined in orange.
