# Supplementary material for: Microbiomes and Planctomycete diversity in large-scale aquaria habitats
Source: PLoS One. 2022 May 12;17(5):e0267881. doi: 10.1371/journal.pone.0267881 (PMC9098025; doi:10.1371/journal.pone.0267881)
Supplement: S3 Fig — The Primer Mapping tool in CLC Genomics Workbench was utilized to map all four primers from the nested PCR onto the assembled 1.3 kb sequence. Primer mapping parameters were set to allow primers to bind to the sequence with a maximum of 10 mismatches and at least 80% coverage. (DOCX) [file pone.0267881.s009.docx]

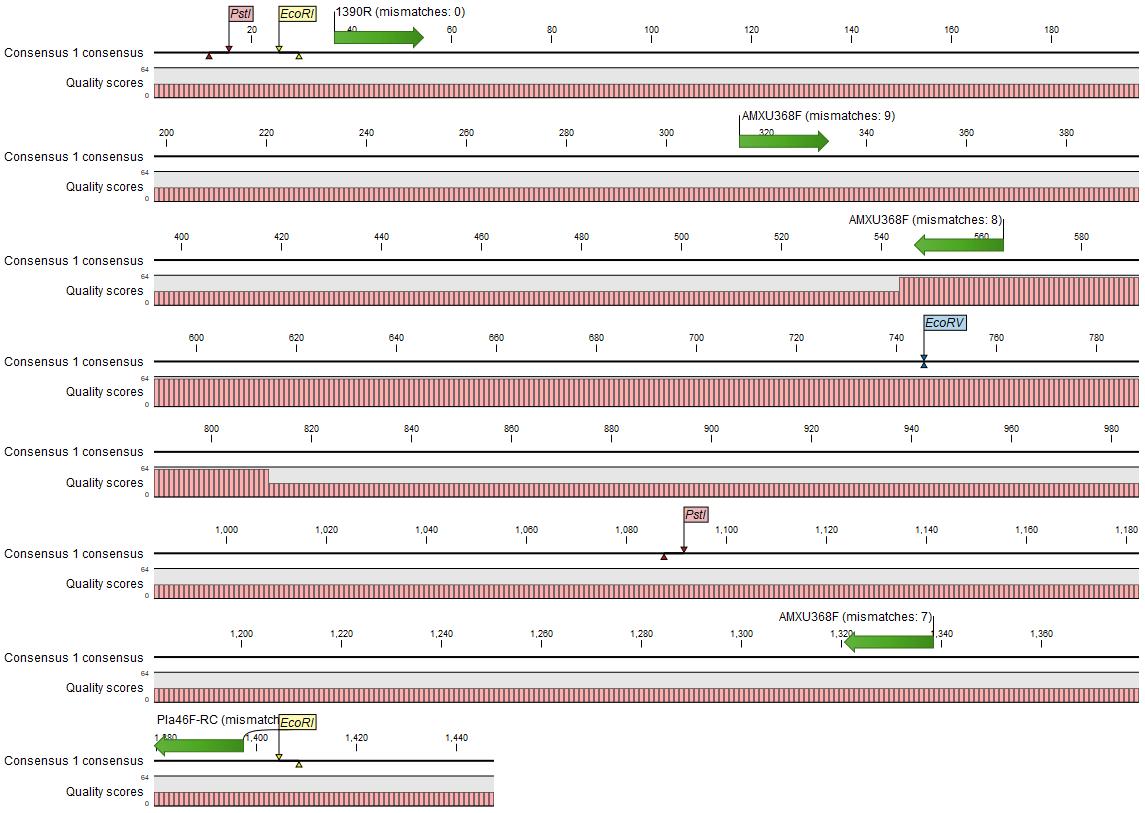


**S3 Figure**. **Diagram showing potential binding sites of primers used in the Planctomycete-targeted nested PCR on a 1.3 kb assembled sequence (T30-CFU-12)**. The Primer Mapping tool in CLC Genomics Workbench was utilized to map all four primers from the nested PCR onto the assembled 1.3 kb sequence. Primer mapping parameters were set to allow primers to bind to the sequence with a maximum of 10 mismatches and at least 80% coverage.
